# The Construction of Surface-Frustrated Lewis Pair Sites to Improve the Nitrogen Reduction Catalytic Activity of In_2_O_3_

**DOI:** 10.3390/molecules28207130

**Published:** 2023-10-17

**Authors:** Mingqian Wang, Ming Zheng, Yuchen Sima, Chade Lv, Xin Zhou

**Affiliations:** 1 Public Teaching Department, Heilongjiang Institute of Construction Technology, Harbin 150025, China; 2 MIIT Key Laboratory of Critical Materials Technology for New Energy Conversion and Storage, School of Chemistry and Chemical Engineering, Harbin Institute of Technology, Harbin150001, China

**Keywords:** In_2_O_3_, N_2_ reduction reaction, density functional theory, surface-frustrated Lewis pairs

## Abstract

The construction of a surface-frustrated Lewis pairs (SFLPs) structure is expected to break the single electronic state restriction of catalytic centers of P-region element materials, due to the existence of acid-base and basic active canters without mutual quenching in the SFLPs system. Herein, we have constructed eight possible SFLPS structures on the In_2_O_3_ (110) surface by doping non-metallic elements and investigated their performance as electrocatalytic nitrogen reduction catalysts using density functional theory (DFT) calculations. The results show that P atom doping (P@In_2_O_3_) can effectively construct the structure of SFLPs, and the doped P atom and In atom near the vacancy act as Lewis base and acid, respectively. The P@In_2_O_3_ catalyst can effectively activate N_2_ molecules through the enzymatic mechanism with a limiting potential of −0.28 eV and can effectively suppress the hydrogen evolution reaction (HER). Electronic structure analysis also confirmed that the SFLPs site can efficiently capture N_2_ molecules and activate N≡N bonds through a unique “donation-acceptance” mechanism.

## 1. Introduction

Ammonia (NH_3_) is not just an irreplaceable nitrogen-containing chemical raw material in the synthesis of traditional chemical products such as fertilizers, nitric acid, and explosives. In recent years, ammonia fuel has emerged as a clean energy source of worldwide interest due to its ease of transport and storage and its lack of carbon emissions in operation [1,2,3]. At present, large-scale industrial ammonia synthesis is still based on the Haber–Bosch (H-B) reaction, which requires high temperatures (400–500 °C) and high pressures (100–200 bar), which not only results in extremely high energy consumption but also in extremely stringent equipment requirements. Many researchers are searching for new and clean ammonia synthesis technology to replace the traditional industrial ammonia synthesis technology in order to alleviate the problems of high resource consumption and severe environmental pollution caused by industrial ammonia synthesis [4]. Among them, electrochemical nitrogen reduction reaction (NRR) technology is a method with great application potential and research value, which has outstanding advantages such as high efficiency, low energy consumption, and simple reaction devices [5,6,7]. Electrochemical nitrogen reduction reaction technology is based on the synthesis of ammonia (or NH_4_^+^) from N_2_ and water (H_2_O or H^+^) at ambient temperature and pressure, driven by electricity with the applied voltage. The conversion and storage of intermittent energy is facilitated when the electricity consumed comes from clean energy sources such as solar, wind, and tidal power.

The development of efficient catalysts is a central task in the commercialization of electrocatalytic nitrogen reduction ammonia technology. It is well known that transition metals (TMs) with both d-orbital electrons and unoccupied d-orbital electronic structures can form N_2_-M σ-donation and M-N_2_ π-bonding configurations with nitrogen molecules, which can lead to the activation of nitrogen via the π-bonding pathway [6]. Therefore, most of the electrocatalysts reported so far contain transition metals such as Fe, Mo, W, etc. However, HER is more likely to occur in most transition metal-based electrocatalysts, leading to problems such as the poor selectivity of the ammonia synthesis reaction on the catalysts and low Faradaic efficiency.

Compared to transition metal catalysts, elemental materials in the p-block are inherently weak in hydrogen precipitation activity, which is feasible for selective electrocatalytic ammonia synthesis. Légaré et al. [8] found that the Lewis acid-containing elemental boron in the boranylidene group can also inject electrons into the N_2_ molecule via back-donation due to the simultaneous presence of empty sp^2^ orbitals and occupied p-orbitals and π-antibonding orbitals to achieve N_2_ activation. By doping porous carbon with elemental fluorine with higher electronegativity, Liu et al. designed and prepared highly active (ammonia yield: 197.7 µg h^−1^ mg_cat_^−1^) p-block elemental-based electrocatalysts (F-doped carbon) enriched with Lewis acid sites and programmed elevated temperature desorption in the nitrogen atmosphere [9]. The N_2_ desorption peak of the F-doped carbon was found to be located at 436 °C, which is 115 °C higher than that of the undoped F sample, indicating that the Lewis acid sites can give the catalyst a stronger nitrogen binding capacity. Hu et al. improved the NRR performance of the catalyst in neutral electrolyte by simultaneously modifying oxygen vacancies and hydroxyl groups on the surface of Bi_4_O_5_I_2_ to induce the creation of unoccupied orbitals around the Bi atoms, which drastically lowered the protonation barriers of N_2_ molecules [10]. The above studies show that the presence of either acidic or basic Lewis sites can, to some extent, improve the ability of the catalyst to interact with N_2_.

The SFLPs system has acidic and basic Lewis active centers that are not quenched with each other, and the construction of this surface structure is expected to break the limitation of the single electronic state of the catalytic center of the p-block elemental materials, which can not only target the inert molecules through the synergistic effect of Lewis acids and bases, but also activate the adsorbed molecules efficiently through the unique “donation-acceptance” process [11]. Lin et al. took advantage of the existence of empty p-orbitals after the sp^2^ hybridization of B atoms and paired electrons after the sp^3^ hybridization of N atoms [12]. The construction of local environments by spatially hindered B-N atom pairs can play a “pulling” role for N_2_ molecules, and inertness can be achieved by weakening the N≡N bond molecular activation by weakening the N≡N bond. This work provides strong support for the construction of SFLPs sites on the surface of p-block element-based electrocatalysts and the elucidation of the mechanism of nitrogen reduction.

Indium-based oxides are a class of p-block elemental materials with simple compositions that are widely used in the catalytic reduction of CO_2_ at room temperature to synthesize CO, CH_3_OH, and other C1 chemicals [13,14,15,16]. The surface atomic arrangement of indium-based oxides is easily tunable, and SFLPs sites can be constructed using simple modifications such as the introduction of oxygen vacancies. Moreover, compared with BCN 2D nanosheets, the SFLPs sites of indium-based oxides can be constructed around the oxygen vacancies on the surface of the material, which also helps to construct high-density and high-activity surface SFLPs sites [17].

In this work, defective In_2_O_3_ with one oxygen vacancy (V-In_2_O_3_) was selected as the research object, and the construction of oxygen defects and heteroatom doping means were used to fine-tune the design of the spatial configuration of the Lewis acid/base center of the SFLPs sites on the surface of the material, the electronic structure, to explore the form of acid/base site combinations in the SFLPs (In/doped elements), and to deeply and systematically investigate the influence of the electronic structure of the doped elements, the spatial site-resistance configuration, and other factors on the FLPs sites on the activation of nitrogen molecules. At the same time, the influence of the constructed SFLPs sites on the nitrogen reduction reaction pathway was analyzed, which provided experimental and theoretical guidance for the preparation of p-region element-based electrocatalysts with excellent performance in nitrogen reduction for ammonia synthesis.

## 2. Results and Discussion

### 2.1. Geometric Structure of Doping V-In_2_O_3_

**Figure 1:**
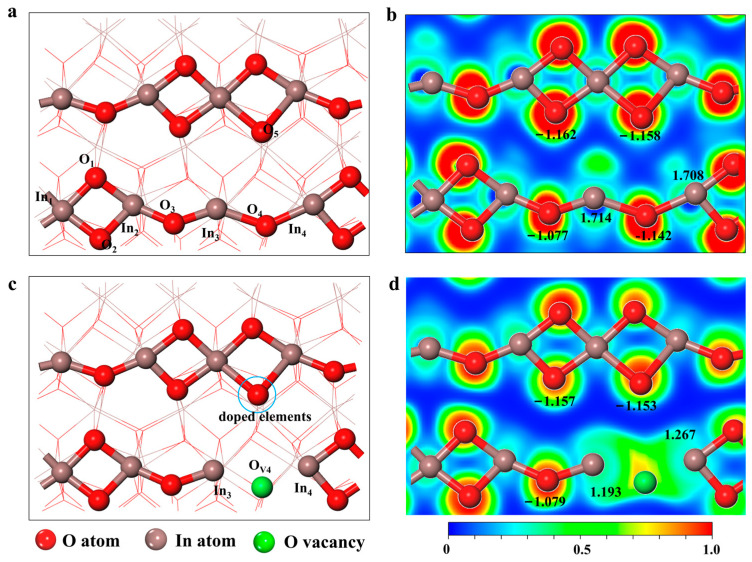
Optimized structure of (**a**) the perfect In_2_O_3_ (110) surface and (**c**) defective In_2_O_3_ (110) with one oxygen vacancy. Electron localization function of (**b**) the perfect In_2_O_3_ (110) surface and (**d**) defective In_2_O_3_ (110) with one oxygen vacancy. The black numbers in the diagram are the Bader charges of the atoms.

The surface of In_2_O_3_ (110) is one of the major exposed crystal planes of c- In_2_O_3_ observed in experiments [18,19,20]. It has also been shown to be thermodynamically stable under electrocatalytic conditions in theoretical studies [21]. Therefore, we chose the surface of In_2_O_3_ (110) for our study. The top layer structure of the optimized perfect In_2_O_3_ (110) surface is shown in Figure 1a, and consists of 8 In atoms and 12 oxygen atoms forming a repeating unit. In addition, previous reports have shown that oxygen vacancies (V_O_) on the surface of In_2_O_3_ can generate SFLPs sites, which serve as active sites for reactions [22,23,24]. At the same time, Qin et al. calculated the vacancy formation energies of different oxygen vacancies using density functional theory, and the calculations showed that the V_O4_ site had the highest formation energy [25].

As shown in Figure 1, in the absence of oxygen vacancies on the surface, In_3_ and In_4_ atoms (Lewis acids) can form localized structures similar to SFLPs with O atoms (Lewis bases) from the other chain, but the O_4_ attached to In_3_ and In_4_ atoms shields them from their functionality as Lewis acids. The unsaturated ligand In atoms (In_4_ and In_5_) and O atoms (O_5_) on the other chain form SFLPs sites when oxygen vacancies are present on the surface of In_2_O_3_ (110) [22,24]. Meanwhile, Bader charge results show that the atomic local charges of In_3_, In_4_, and O_5_ are +1.193 and +1.267, −1.153, respectively. The results show that the creation of oxygen vacancies makes two In atoms (In_3_ and In_4_) on the In_2_O_3_(110) surface into potential electron donors, further suggesting that oxygen vacancy defects can construct SFLPs sites compared to a perfect In_2_O_3_(110) surface. However, the O_4_ vacancy defects on the surface of In_2_O_3_(110) still hold a small charge, which has a shielding effect on the catalytic ability of SFLPs, as can be seen from the electron localization function diagram in Figure 1d.

**Figure 2:**
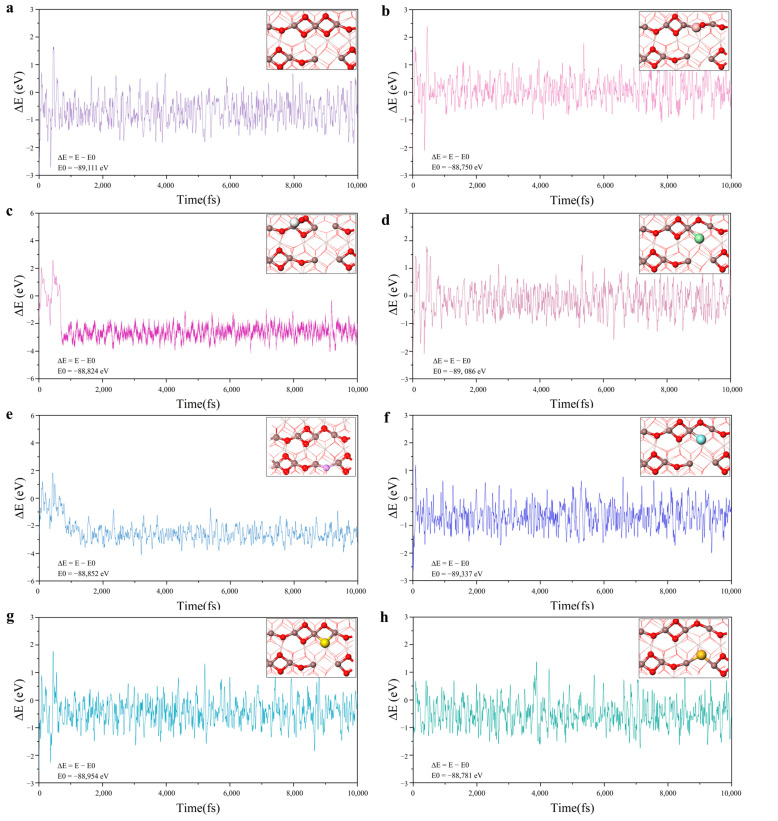
Variations in temperature and energy against the time for the AIMD simulations of (**a**) V-In_2_O_3_, (**b**) B@In_2_O_3_, (**c**) C@In_2_O_3_, (**d**) Cl@In_2_O_3_, (**e**) F@In_2_O_3_, (**f**) P@In_2_O_3_, (**g**) S@In_2_O_3_, and (**h**) Si@In_2_O_3_. The simulation was run under 400 K for 10ps with a time step of 1 fs.

Indium oxide is a kind of P region elemental material with simple composition which is often used in the catalytic reduction of CO_2_ at room temperature to synthesize C1 chemicals such as CO and CH_3_OH. The surface atomic structure of indium-based oxides is easily modified, such as by heteroatom doping to build SFLPs sites. Meanwhile, as far as we know, there have been no investigations on the construction of SFLPs by doping nonmetallic elements on the surface of In_2_O_3_. To further enhance the catalytic ability of the SFLPs sites and avoid the shielding effect of oxygen vacancies in different electronic states, we modified the surface by replacing O_5_ with various nonmetal elements (named n-M@In_2_O_3_, nonmetal = C, N, F, Si, P, S, Cl,) and screened for efficient and stable acid-base site combinations in SFLPs. The optimized structure of several nonmetal elements doped in In_2_O_3_ (110) is shown in Figure S1. The results of the geometry optimization showed that the different non-metallic elements selected could be used to replace the O_5_ atom for the modification of the SFLPs site. Subsequently, the stability of the constructed SFLPs site at the actual applied temperature was further tested using 10 ps AIMD simulation for all doped structures. As shown in Figure 2, the curves of the total energy change of the different structures were calculated as the simulation time increased. For C@In_2_O_3_ and F@In_2_O_3_, the total energy of the system became significantly smaller within the first 1ps of the simulation. Correspondingly, the structure of the surface SFLPs sites changed. The C atom was oxidized to CO and then left the surface, while the F atom moved to the O vacancy and combined with In_3_ and In_4_ atoms, also changing the surface SFLPs site structure. Furthermore, although the total energy of B@In_2_O_3_ and Si@In_2_O_3_ did not change significantly during the simulation, the SFLPs site structure changed. The doped B atoms bound to the oxygen atoms on the surface to break the SFLPs site structure, while the Si atoms filled the original O_4_ vacancy defect, as well as the doped F atoms. The diagram of the radial distribution function also supported the above results, as can be seen in Figure S2. In general, AIMD simulation results showed that only the doping and substitution of P, S, and Cl atoms could construct relatively stable SFLPs site structures. Therefore, in the following discussion, P@In_2_O_3_, S@In_2_O_3_, and Cl@In_2_O_3_ were selected as research objects to further screen potential and efficient NRR catalysts.

### 2.2. Adsorption and Protonation of Nitrogen

**Figure 3:**
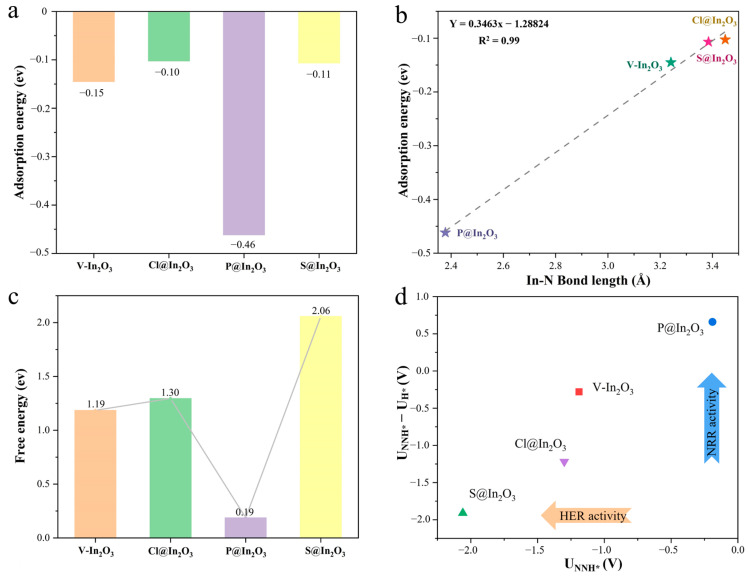
(**a**) Adsorption energy of N_2_ on different materials. (**b**) The adsorption energy of N_2_ on different materials is a function of the In-N bond length. (**c**) The free energy of the first hydrogenation of N_2_ on different materials. (**d**) Diagram of the potential difference between the NRR and HER on different materials.

N_2_ adsorption is the first and most essential step of the whole NRR process. As shown in Figure S3, the most stable structure of N_2_ adsorbed on V-In_2_O_3_, P@In_2_O_3_, S@In_2_O_3_, and Cl@In_2_O_3_ was optimized. The results show that among the four materials, N_2_ could be stably adsorbed only on the P@In_2_O_3_ surface. Simultaneously, the bond length data of N_2_ on different materials were summarized in Table S1. The length of the adsorbed N-N bond on P@In_2_O_3_ was 1.17 Å, while the length of the free N_2_ bond was 1.108 Å, indicating that the N-N bond was activated. In addition, the adsorption energy of N_2_ on different materials was calculated, as shown in Figure 3a. The adsorption energies of N_2_ on V-In_2_O_3_, P@In_2_O_3_, S@In_2_O_3_, and Cl@In_2_O_3_ were −0.15, −0.10, −0.46, and −0.11 eV, respectively. The more negative the adsorption energy, the stronger the adsorption of N_2_ on the catalysts. The results further showed that P@In_2_O_3_ can effectively adsorb N_2_ molecules. It was also interesting to find that there was a good linear relationship between the bond length of In-N and the adsorption energy of N_2_, where the shorter the bond length, the stronger the adsorption.

Following the capture and adsorption of N_2_ molecules, the first hydrogenation step of N_2_* is critical and is often the potential determining step (PDS) in the protonation step. In addition, the Gibbs free energy (ΔG) of the first hydrogenation of N_2_* is often used as a descriptor for selecting catalysts with excellent catalytic performance. Therefore, we calculated the ΔG values of N_2_*→N_2_H* on V-In_2_O_3_, P@In_2_O_3_, and S@In_2_O_3_. Following the capture and adsorption of N_2_ molecules, the first hydrogenation step of N_2_* is critical and is often the potential determining step (PDS) in the protonation step. In addition, the Gibbs free energy (ΔG) of the first hydrogenation of N_2_* is often used as a descriptor for selecting catalysts with excellent catalytic performance. Therefore, we calculated the ΔG values of N_2_*→N_2_H* on V-In_2_O_3_, P@In_2_O_3_, S@In_2_O_3_, and Cl@In_2_O_3_ to screen potential NRR catalysts. As can be seen in Figure 3c, the results of the ΔG calculations show that the protonation of N_2_* on the surfaces of V-In_2_O_3_, S@In_2_O_3_, and Cl@In_2_O_3_ is extremely difficult. However, the free energy of the protonation of N_2_* on the surface of P@In_2_O_3_ is only 0.19 eV, which indicates that P@In_2_O_3_ can effectively activate the N-N triple bonds and lower the energy barrier of the protonation of N_2_*. Therefore, P@In_2_O_3_ is selected as a candidate NRR electrocatalyst for further investigation. Under the real conditions of electrocatalysis (in aqueous solution), HER is inevitable as the main side reaction of the NRR system [5,6,7]. As show in Figure S4, the ΔG values of HER for V-In_2_O_3_, P@In_2_O_3_, S@In_2_O_3_, and Cl@In_2_O_3_ are −0.91, −0.85, 0.15, and −0.08 eV, respectively. In addition, the potential difference between the N_2_* protonation and HER processes (U_NNH*_ − U_H*_) was calculated to evaluate the selectivity of electrocatalysis reactions with different catalysts, as shown in Figure 3d. The results show that P@In_2_O_3_ has excellent catalytic selectivity for NRR and can effectively inhibit the HER, which is considered to be a promising electrocatalyst for NRR.

### 2.3. Catalytic Mechanism in Solution

**Figure 4:**
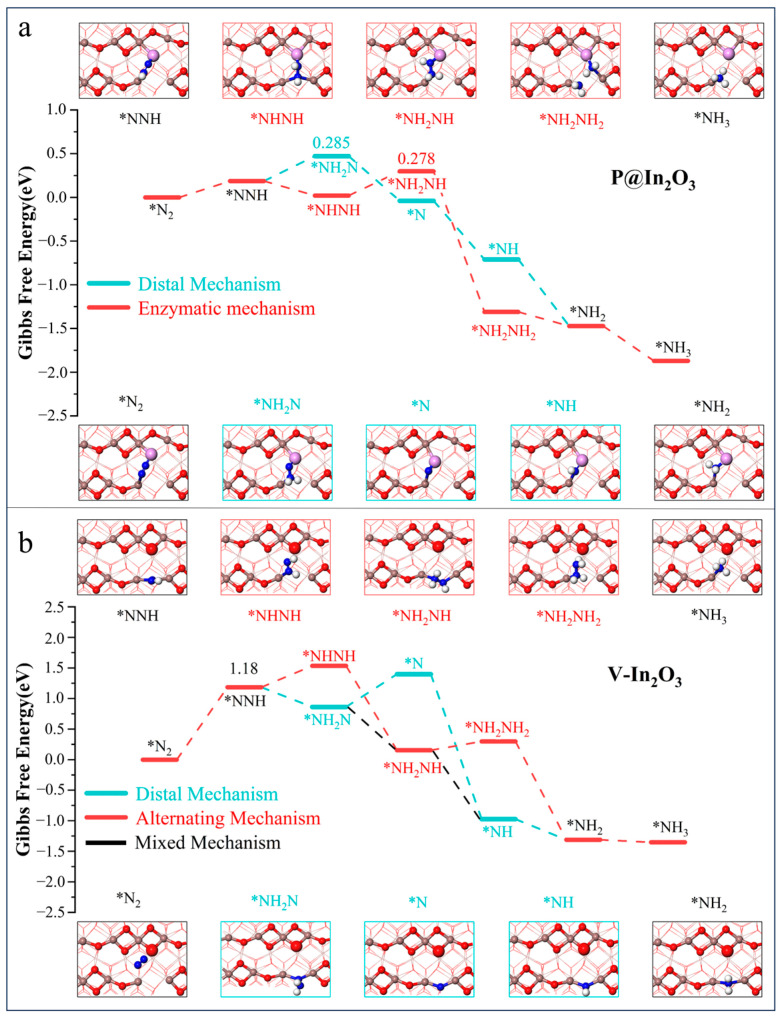
Gibbs free energy diagrams and intermediate structure of N_2_ reduction process on (**a**) P@In_2_O_3_ and (**b**) V-In_2_O_3_.

To explore the reaction mechanism of NRR on P@In_2_O_3_, Gibbs free energies of different mechanisms on P@In_2_O_3_ and V-In_2_O_3_ were calculated and the results are shown in Figure 4. The implicit solvent model was used to consider the influence of the solvation effect on the reaction when calculating the Gibbs free energy diagram. The adsorption and desorption processes are not sensitive to the solvation effect [26], so the calculation started from N_2_* protonation. The possible NRR pathway on P@In_2_O_3_ is shown in Figure 4a, and since N_2_* is adsorbed on the surface of P@In_2_O_3_ in a side-on configuration, the enzymes and distal mechanisms were mainly considered for the NRR process. The results show that the PDS of the enzyme mechanism was NHNH* + H^+^/e^−^ →NH_2_NH* and ΔG was 0.278 eV, where the PDS of the distal mechanism was NHN*+ H^+^/e^−^ → NH_2_N* with ΔG of 0.285 eV. It was found that the PDS of the above two mechanism species was not the typical first hydrogenation process, and the protonation steps after PDS were spontaneous processes. At the same time, the limiting potential difference between the two mechanisms was only 0.07 eV, suggesting that NRR can occur on P@In_2_O_3_ through both the enzyme and distal mechanisms.

As shown in Figure 4b, the N_2_ molecule was adsorbed in an end-on configuration on the surface of V-In_2_O_3_, so the alternating, distal, and mixed mechanisms are considered. The results show that the PDS of these three reaction mechanisms was the first hydrogenation process (N_2_*+ H^+^/e^−^→NNH*) with the ΔG is 1.18 eV. In the case of the mixing mechanism, the protonation steps after the PDS were all processes of energy decrease and could be formed spontaneously. Meanwhile, for the other two mechanisms, the protonation steps after the formation of the first NH_3_ were all spontaneous processes. It is noteworthy that the NNH* intermediate was not adsorbed at the constructed SFLPs site. Instead, it formed In-N bonds with the two In atoms (In_3_ and In_4_) at the oxygen vacancy and was adsorbed on the surface of V-In_2_O_3_ by end-on configuration. This also shows that it is not sufficient to build SFLPs sites by oxygen vacancies alone.

### 2.4. Origin of Catalytic Activity

**Figure 5:**
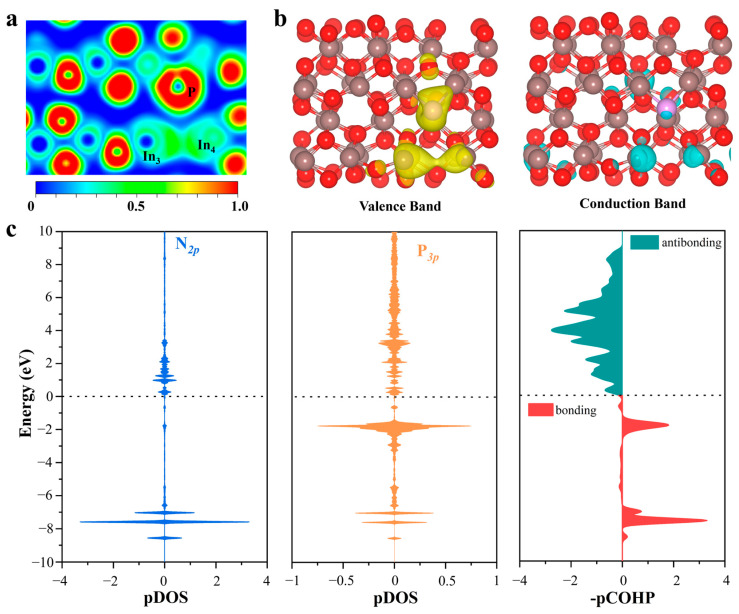
(**a**) Electron localization function and (**b**) partial charge density of the VB and CB of P@In_2_O_3_. The value of the isosurface is 0.002 e/Å^−3^. (**c**) The pDOS and -pCOHP between N and P atoms on P@In_2_O_3_-adsorbed N_2_.

In order to identify the origin of catalytic activity at the P-doped SFPLs sites on P@In_2_O_3_, the ELF levels of V-In_2_O_3_, P@In_2_O_3_, S@In_2_O_3_, and Cl@In_2_O_3_ were calculated and the results are shown in Figure 5 and Figure S6. It can be clearly seen from the figure that there are obvious localization electrons at the oxygen vacancy (between In_3_ and In_4_) on the surfaces of V-In_2_O_3_, S@In_2_O_3_, and Cl@In_2_O_3_, while the electron density of the oxygen vacancy on P@In_2_O_3_ equals the uniform density of the electron gas. These oxygen vacancies with localization electrons shield the catalytic activity of the constructed SFLPs sites by making it difficult to induce empty orbitals (Lewis acids) from the surrounding metal atoms. Interestingly, the results of partial charge density calculations for the valence band (VB) and conduction band (CB) support this opinion. The partial charge density values of the VB and CB of V-In_2_O_3_, P@In_2_O_3_, S@In_2_O_3_, and Cl@In_2_O_3_ are shown in Figure S7. It was found that only the VB of P@In_2_O_3_ was mainly contributed by the In atoms (In_3_ and In_4_) and the P atoms, whereas the VB of the other results was mainly contributed by the In_3_ and In_4_ atoms. As the major constituent in VB, the electron-rich P atom acts as a Lewis base site. It can interact with the empty π orbital of N_2_, providing electrons to activate N_2_ molecules. In the meantime, the CB of P@In_2_O_3_ is mainly contributed by the In_3_ atoms. The empty orbital of the In_3_ atom acts as a Lewis acid site and is able to accept the electron of the N_2_ molecules. The apparent electron “donation-acceptance” process between the N_2_ molecules and the SFLPs sites on P@In_2_O_3_ promotes the effective activation of the N≡N bonds.

In order to verify the “donation-acceptance” process of the electrons, the difference charge density difference of the N_2_ adsorbed by the different structures was calculated, as shown in Figure S8. The results show that electron transfer to N_2_ occurred at the P@In_2_O_3_ SFLPs site, which confirms the “donation-acceptance” mechanism of electrons. The Bader charge analysis also confirms this phenomenon and the calculated values in Table S3 indicate that the In_3_ and P atoms are injected into the N_2_* molecule. In addition, the integrals of projected crystal orbital Hamilton population (ICOHP) data and bond length data suggest that the SFPLs sites can effectively activate the N≡N bonds, as shown in Tables S1 and S4. At the same time, the partial densities of states (PDOS) and the projected crystal orbital Hamilton population (pCOHP) of N_2_ adsorbed on different structures were calculated to further investigate the “donation-acceptance” mechanism. As shown in Figure 5c, the *2p* orbital of N_2_ and the *3p* orbital of P have a large hybrid region at the high-energy region (−2 eV), and a 2π* orbital of N_2_ shift to the high-energy region near the Fermi level, indicating strong electron donation interaction and orbital interaction between the P atom and N_2_. As shown in Figure S9, the bonding orbitals are mainly contributed by the mutual hybridization of the *s* and *p* orbitals of the In_3_ and N atoms between the −6 and −8 eV levels. This shows that the empty *5p* orbital of the In atom can effectively accept the σ electron of the N_2_ molecule and activate the N≡N bonds.

## 3. Materials and Methods

The DS-PAW program integrated into the Device Studio program [27] was used to perform the calculations under the framework of density functional theory (DFT). The interaction between valence electrons and the ionic core was described using the projection-augmented wave (PAW) base set [28,29], within the exchange-correlation function described by Perdew, Burke, and Ernzerhof (PBE) [30]. The D3 correction method proposed by Grimme [31,32] was used to make up for the deficiency of the GGA functional description of van der Waals interaction. One-electron Kohn−Sham orbitals were expanded with a kinetic energy cutoff of 630 eV. The geometry optimization adopted the 3 × 2 × 1 Monkhorst-Pack grid of the K-points mesh to sample the Brillouin zone. The convergence criterion for self-consistent iteration was set to 1 × 10^−5^ eV and the structures were fully relaxed until the final force on each atom was less than 0.03 eV Å^−1^. To test the stability of the doped structures, Ab initio molecular dynamics (AIMD) simulations of all doping systems were performed using a canonical ensemble (NVT) with Nosé thermostat at 400 K temperature with a total time of 10 ps and a time step of 1 fs. In addition, the density of states (DOS) was calculated using the Vienna Ab initio Simulation Package (VASP) [33,34]. DOS calculations using 4 × 3 × 1 Monkhorst-Pack grids of k-points mesh. At the same time, the LORBSTER [35] program was used to analyze the properties of COHP [36].

In this work, the most thermodynamically stable body-centered cubic bixbyite (c-In_2_O_3_) crystal structure was selected, which had undergone extensive theoretical and experimental investigation [19,37,38]. The surface of In_2_O_3_ (110) was modeled in a (1 × $\sqrt{2}$) supercell with five periodic atomic layers containing 40 In atoms and 60 O atoms. The bottom three layers of the model were fixed to simulate bulk properties, while the rest of the model was allowed to relax during the geometry optimization process. A vacuum gap of 15 Å was set for all calculations to avoid periodic interactions between the images. The implicit solvent model was used to calculate the Gibbs free energy to consider the solvation effect, and the dielectric constant was set to 78.5 to simulate the aqueous environment.

## 4. Conclusions

In this study, a series of SFPLs site structures were constructed on the surface of In_2_O_3_ by means of a heteroatom doping strategy. Among the eight different structures, P@In_2_O_3_, with a stable structure and high catalytic activity, was selected for the electrochemical synthesis of ammonia. The P@In_2_O_3_ catalyst could effectively reduce N_2_ molecules by both enzymatic and distal mechanisms, and it was found that neither mechanism of PDS was a typical first hydrogenation process. Electronic structure analysis shows that the P doping successfully constructed SFPLs sites on the surface of In_2_O_3_ and that the In and P atoms were Lewis acid sites and base sites, respectively. The electron-rich P atom (Lewis bases) could interact with the empty π orbital of N_2_, donating electrons to activate the N_2_ molecule. Meanwhile, the In atoms with empty orbitals (Lewis acid) could accept the σ electron of the N_2_ molecule. In summary, this study provides new insights into the reasonable regulation and improvement of the surface structure of P-region elementary materials and provides theoretical guidance for the construction of SFLPs sites.

## Data Availability

Not applicable.

## Supplementary Materials

The following supporting information can be downloaded at: https://www.mdpi.com/article/10.3390/molecules28207130/s1, Figure S1: Optimized structure of (a) perfect In_2_O_3_ (110), (b) V-In_2_O_3_, (c) B@V-In_2_O_3_, and (d) C@V-In_2_O_3_, (e) Cl@V-In_2_O_3_, (f) F@V-In_2_O_3_, (g) P@V-In_2_O_3_, (h) S@V-In_2_O_3_, (i) Si@V-In_2_O_3_; Figure S2: Radial distribution function of (a) perfect In_2_O_3_ (110), (b) V-In_2_O_3_, (c) B@V-In_2_O_3_, and (d) C@V-In_2_O_3_, (e) Cl@V-In_2_O_3_, (f) F@V-In_2_O_3_, (g) P@V-In_2_O_3_, (h) S@V-In_2_O_3_, (i) Si@V-In_2_O_3_; Figure S3: Optimized structure of N_2_ adsorbed on (a) V-In_2_O_3_, (b) P@V-In_2_O_3_, (c) S@V-In_2_O_3_, and (d) Cl@V-In_2_O_3_; Figure S4: The free energy diagram of the HER on different materials. Figure S5: The optimized structure of the HER on (a) V-In_2_O_3_, (b) P@V-In_2_O_3_, (c) S@V-In_2_O_3_, and (d) Cl@V-In_2_O_3_; Figure S6: Electron Localization Function of (a) V-In_2_O_3_, (b) P@V-In_2_O_3_, (c) S@V-In_2_O_3_, and (d) Cl@V-In_2_O_3_; Figure S7: The Partial charge density of the VB and CB of (a) V-In_2_O_3_, (b) P@V-In_2_O_3_, (c) S@V-In_2_O_3_, and (d) Cl@V-In_2_O_3_; Figure S8: The chare density difference of N_2_ adsorbed on (a) V-In_2_O_3_, (b) P@V-In_2_O_3_, (c) S@V-In_2_O_3_, and (d) Cl@V-In_2_O_3_; Figure S9: The -pCOHP between N and In_3_ atom on P@In_2_O_3_ adsorbed N_2_. Table S1: The partial bond length (Å) of N_2_ adsorbed on the V-In_2_O_3_, P@V-In_2_O_3_, S@V-In_2_O_3_, and Cl@V-In_2_O_3_ ;Table S2: The partial Bader charge of the V-In_2_O_3_, P@V-In_2_O_3_, S@V-In_2_O_3_, and Cl@V-In_2_O_3_; Table S3: The partial Bader charge of N_2_ adsorbed on the V-In_2_O_3_, P@V-In_2_O_3_, S@V-In_2_O_3_, and Cl@V-In_2_O_3_; Table S4: The partial Integrals of pCOHP data (-∫pCOHP) of N_2_ adsorbed on the V-In_2_O_3_, P@V-In_2_O_3_, S@V-In_2_O_3_, and Cl@V-In_2_O_3_.
