# Supplementary material for: The Construction of Surface-Frustrated Lewis Pair Sites to Improve the Nitrogen Reduction Catalytic Activity of In2O3
Source: Molecules. 2023 Oct 17;28(20):7130. doi: 10.3390/molecules28207130 (PMC10608886; doi:10.3390/molecules28207130)
Supplement: Supplementary file 1 [file molecules-28-07130-s001.zip › molecules-2660726-supplementary.pdf]

Construction of Surface Frustrated Lewis Pair sites to improve the nitrogen reduction catalytic activity of In_2O_3

Mingqian Wang [1,2†], Ming Zheng[2†], Yuchen Sima[2], Chade Lv [2*], Xin Zhou[2*]

[1]Public Teaching Department, Heilongjiang Institute of Construction Technology, Harbin 150000, China

[2]MIIT Key Laboratory of Critical Materials Technology for New Energy Conversion and Storage, School of Chemistry and Chemical Engineering, Harbin Institute of Technology, Harbin150001 P. R. China

*Corresponding authors: lv.chade@hlju.edu.cn and zhoux@hit.edu.cn

†The authors contributed equally to this work.

Computational methods and model

Free energy corrections

In searching for possible thermodynamic routes, all free energies (ΔG) were calculated by the following equation:

$\Delta G = \Delta E_{DFT} + \Delta ZPE - T\Delta S$

where ΔE_{DFT} is the energy difference between reactants and products, obtained from DFT calculations; ΔZPE and ΔS are the energy differences in zero-point energy and entropy; T is 298.15 K. ZPE of all adsorbates were calculated from Vibrational analysis, limited to the surface species and keeping the rest of the system fixed, was carried out by calculating the Hessian matrix with a finite difference approach with a step size of 0.015 Å. Vibrational contributions to the entropies for substrate were considered to be inconsequential and counted as zero in the part of TΔS. For molecules, the vibrational contributions to the entropies were calculated from standard thermodynamic tables at 298.15 K and 1 atm. The proton electron pairs transfer energy involved in the reaction pathway is solved by using the computational hydrogen electrode model.

Adsorption energy

Various intermediates adsorbed on different sites on the surface were considered. Generally, the adsorption sites can be classified into three types: top, bridge, and hollow sites. Hollow site includes fcc-like and hcp-like sites in close-packed crystal structure.

Adsorption energy is calculated by the following equation:

$$\Delta E_{ads} = E_{adsorbate\text{-}slab} - E_{adsorbate} - E_{slab}$$

$E_{adsorbate\text{-}slab}$ is the total energy of molecules or intermediates adsorbed on the slab surface, $E_{adsorbate}$ and E_{slab} are energies for the isolated adsorbate and slab, respectively.

Figures

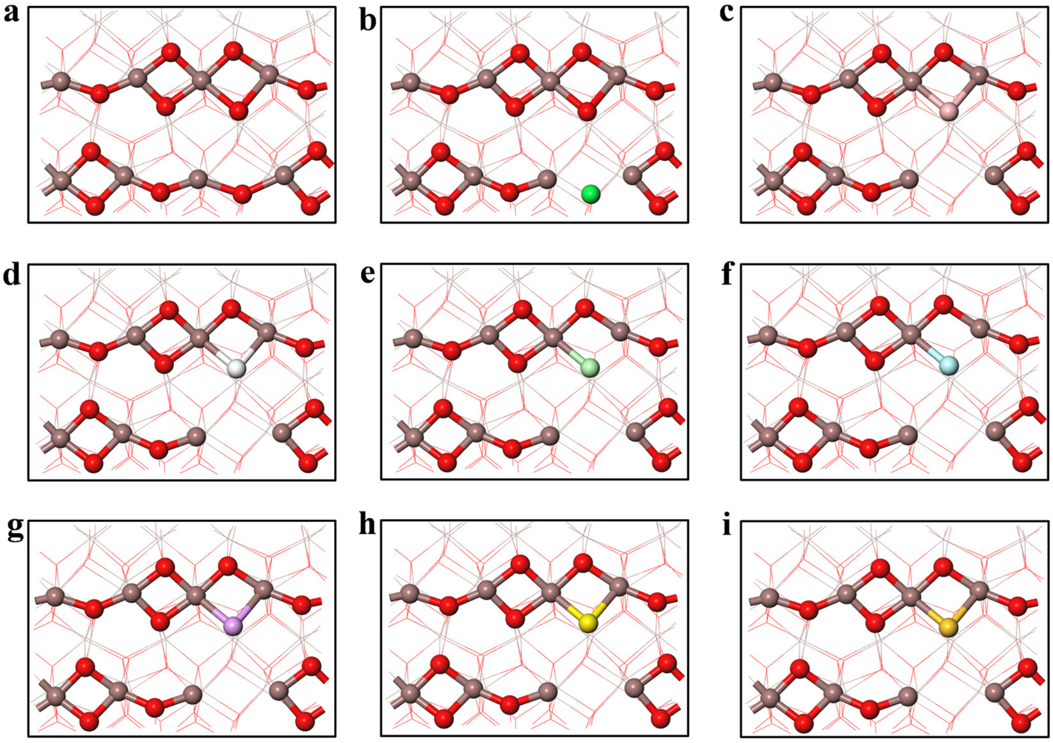

Figure S1 Optimized structure of (a) perfect In_2O_3 (110), (b) V-In_2O_3, (c) B@V-In_2O_3, and (d) C@V-In_2O_3, (e) Cl@V-In_2O_3, (f) F@V-In_2O_3, (g) P@V-In_2O_3, (h) S@V-In_2O_3, (i) Si@V-In_2O_3.

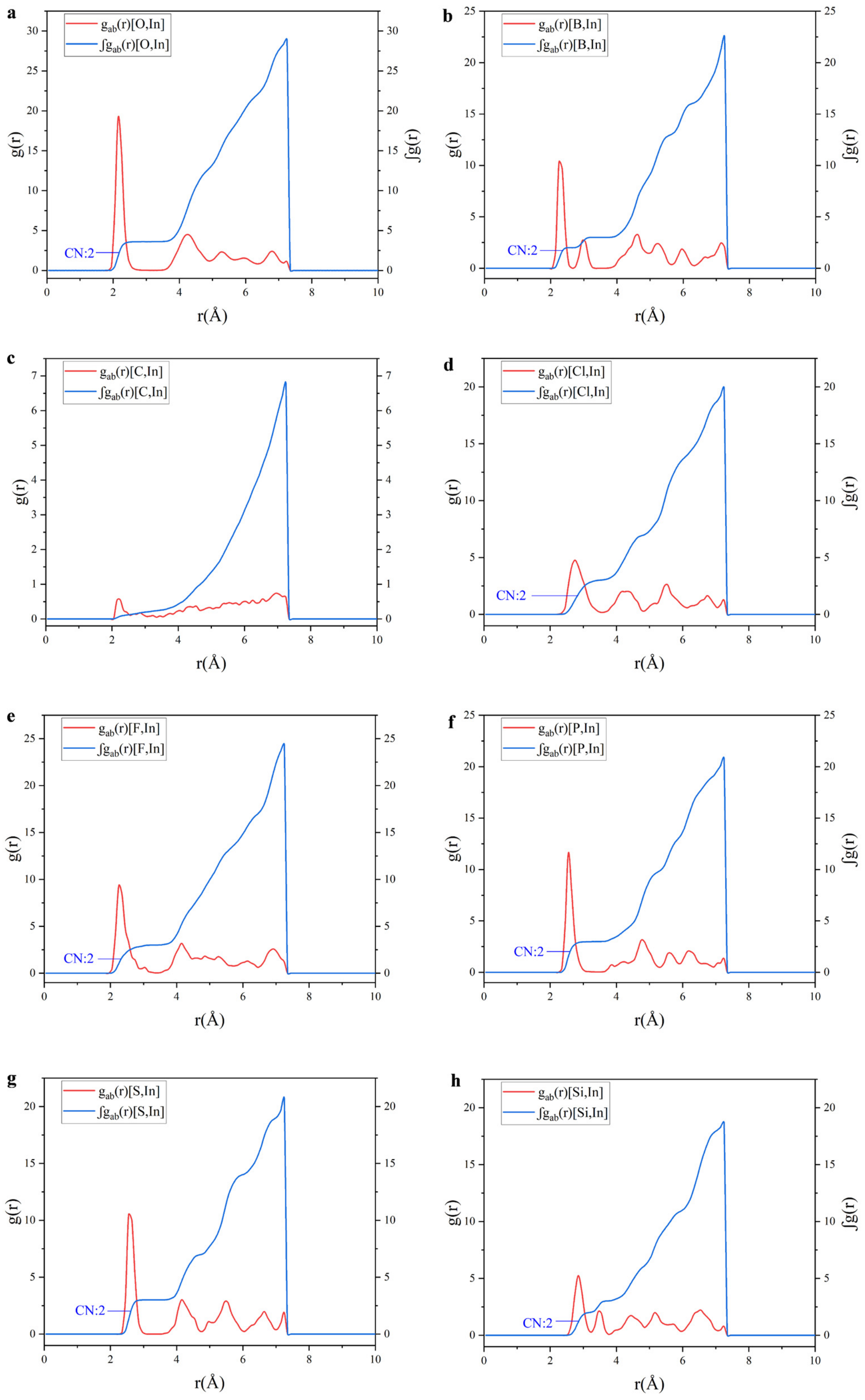

Figure S2 Radial distribution function of (a) V-In_2O_3 (110), (b) B@V-In_2O_3, (c) C@V-In_2O_3, and (d) Cl@V-In_2O_3, (e) F@V-In_2O_3, (f) P@V-In_2O_3, (g) S@V-In_2O_3, (h) Si@V-In_2O_3

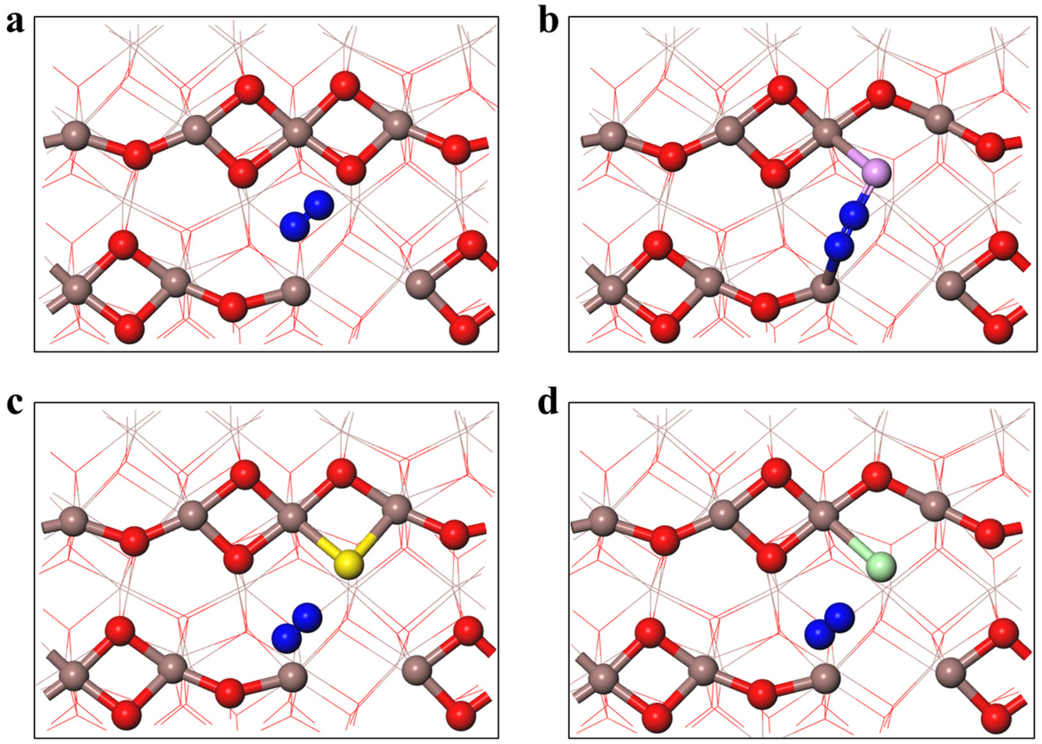

Figure S3 Optimized structure of N_2 adsorbed on (a) V-In_2O_3, (b) P@V-In_2O_3, (c) S@V-In_2O_3, and (d) Cl@V-In_2O_3.

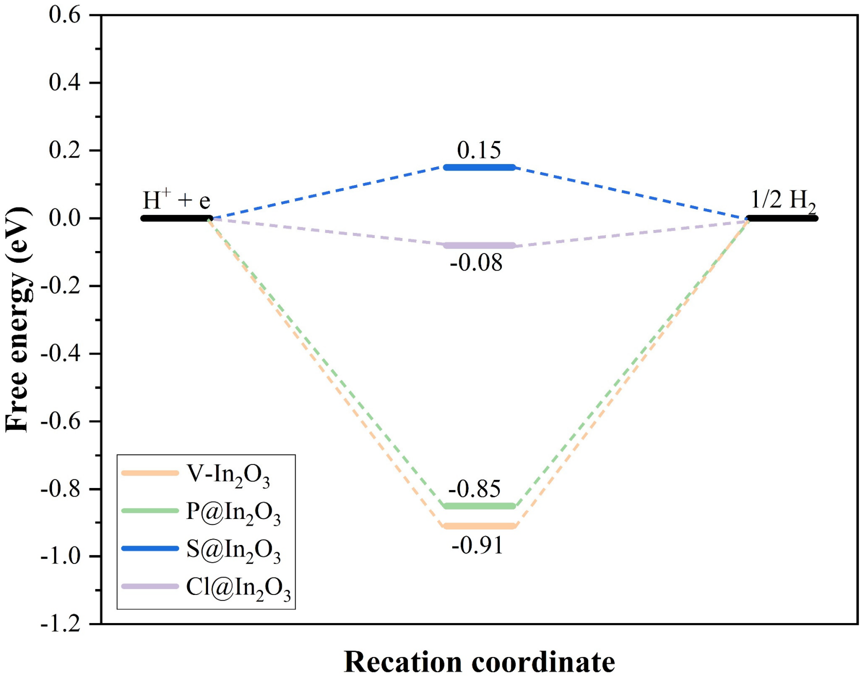

Figure S4 The free energy diagram of the HER on different materials.

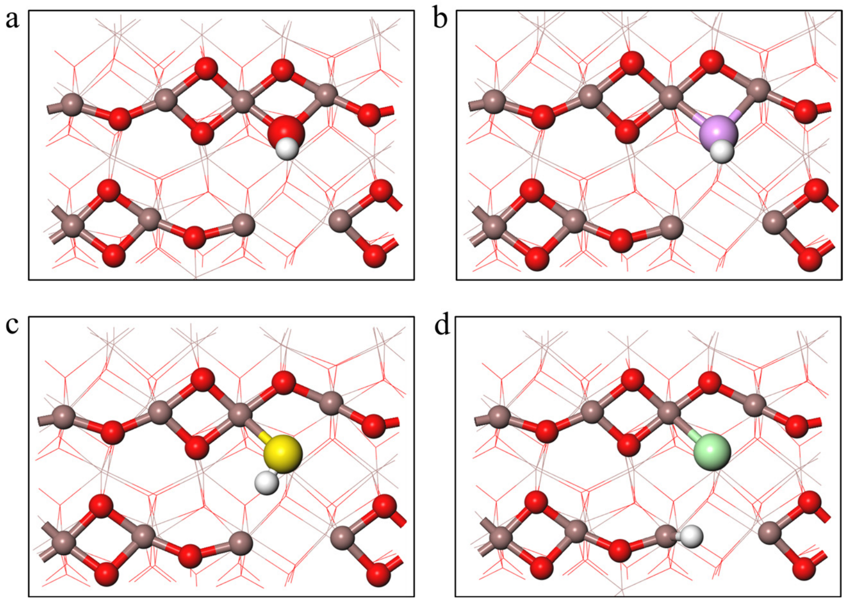

Figure S5 The optimized structure of the HER on (a) V-In_2O_3, (b) P@V-In_2O_3, (c) S@V-In_2O_3, and (d) Cl@V-In_2O_3.

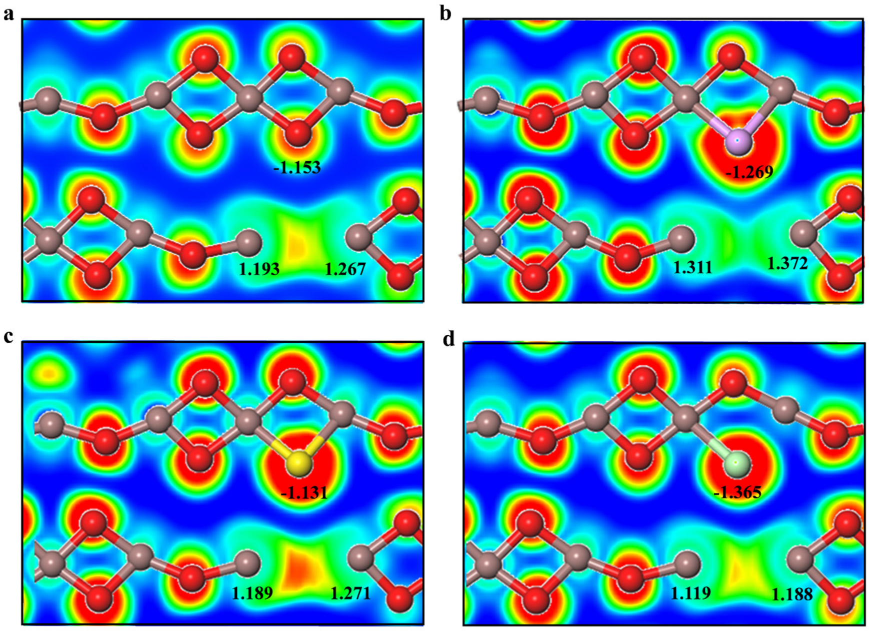

Figure S6. Electron Localization Function of (a) V-In_2O_3, (b) P@V-In_2O_3, (c) S@V-In_2O_3, and (d) Cl@V-In_2O_3. The black numbers in the diagram are the Bader charges of the atoms.

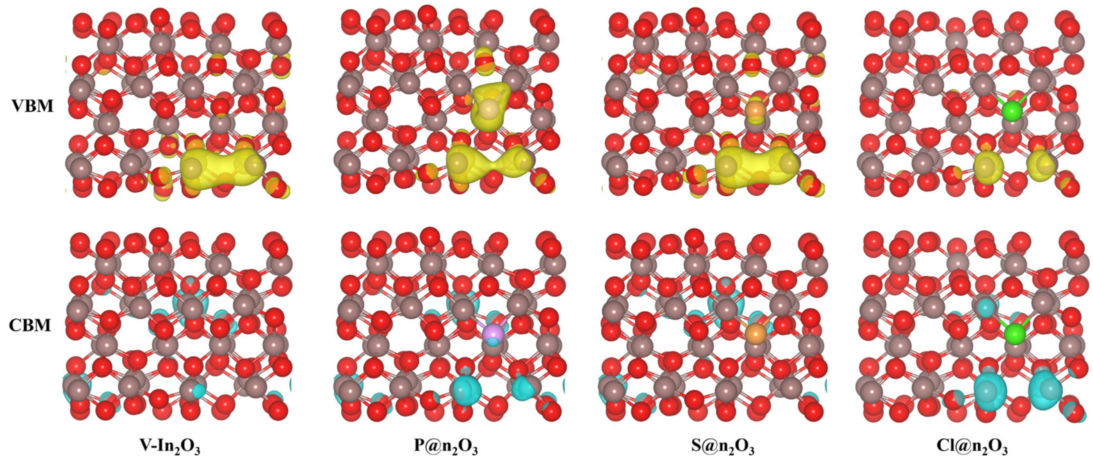

Figure S7. The Partial charge density of the VB and CB of (a) V-In_2O_3, (b) P@V-In_2O_3, (c) S@V-In_2O_3, and (d) Cl@V-In_2O_3. The value of isosurface is 0.002 e/Å $^{-3}$

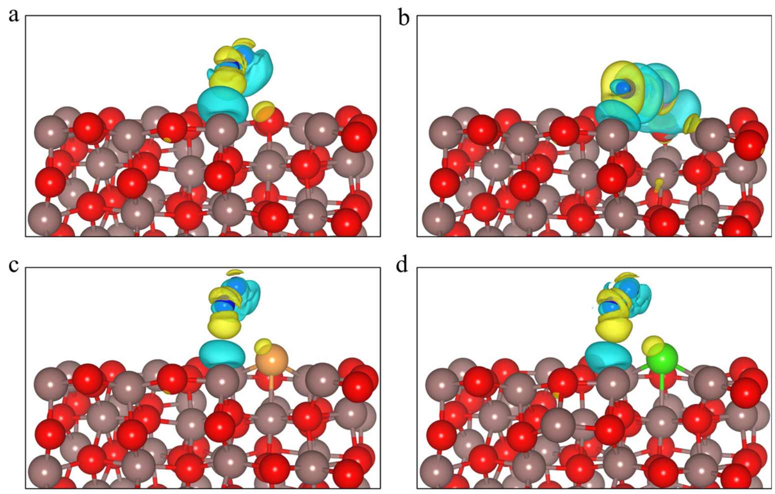

Figure S8. The chare density difference of N_2 adsorbed on (a) V-In_2O_3, (b) P@V-In_2O_3, (c) S@V-In_2O_3, and (d) Cl@V-In_2O_3. The value of isosurface is 0.002 e/Å $^{-3}$

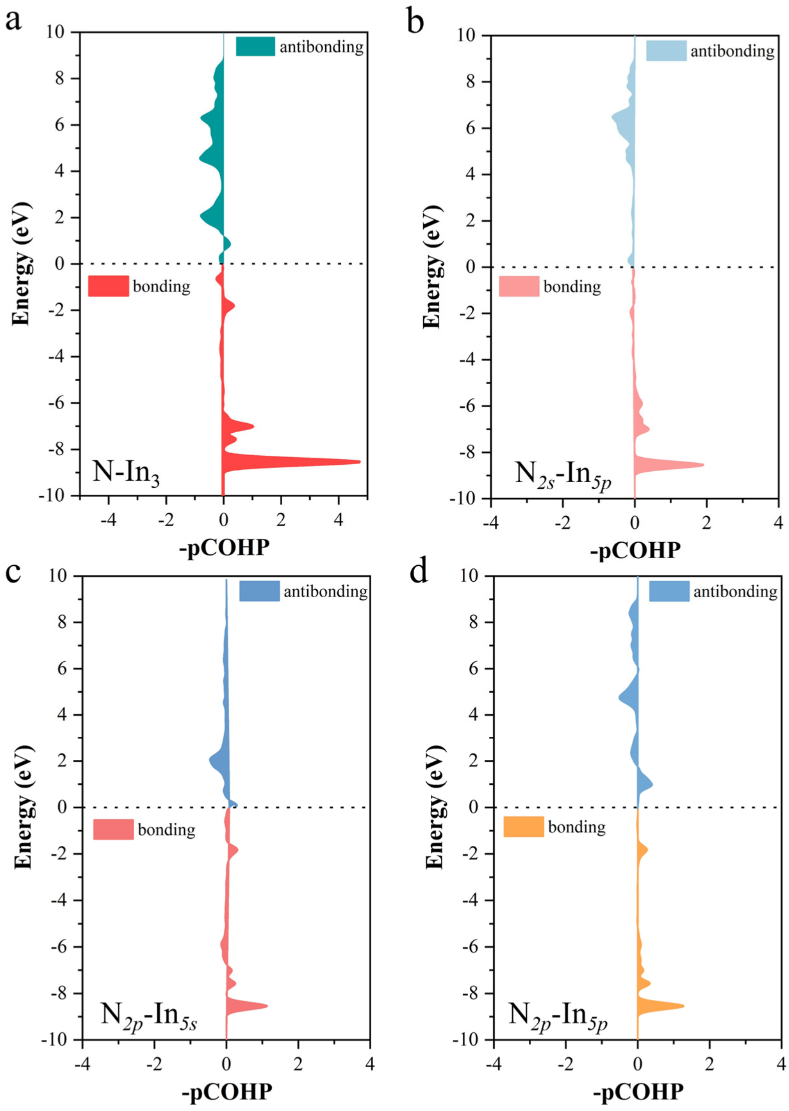

Figure S9 The -pCOHP between N and In_3 atom on $P@In_2O_3$ adsorbed N_2.

Tables

Table S1 The partial bond length (Å) of N_2 adsorbed on the V-In_2O_3, P@V-In_2O_3, S@V-In_2O_3, and Cl@V-In_2O_3.

Bond	V-In_2O_3	P@V-In_2O_3	S@V-In_2O_3	Cl@V-In_2O_3
N-N	1.10	1.10	1.17	1.10
N-In	3.24	3.39	2.38	3.45
N-O/S/P/Cl	3.26	3.92	1.62	3.73

Table S2 The partial Bader charge of the V-In_2O_3, P@V-In_2O_3, S@V-In_2O_3, and Cl@V-In_2O_3.

Atom	V-In_2O_3	P@V-In_2O_3	S@V-In_2O_3	Cl@V-In_2O_3
In_3	1.19	1.19	1.31	1.12
In_4	1.27	1.27	1.19	1.19
O/S/P/Cl	-1.15	-1.13	-1.27	-1.36

Table S3 The partial Bader charge of N_2 adsorbed on the V-In_2O_3, P@V-In_2O_3, S@V-In_2O_3, and Cl@V-In_2O_3.

Atom	V-In_2O_3	P@V-In_2O_3	S@V-In_2O_3	Cl@V-In_2O_3
N	-0.05	-0.13	-0.03	-0.03
N	0.02	-0.74	0.01	0.01
In	1.24	1.48	1.23	1.15
O/S/P/Cl	-1.15	0.39	-0.87	-0.63

Table S4 The partial Integrals of pCOHP data (-∫pCOHP) of N_2 adsorbed on the V-In_2O_3, P@V-In_2O_3, S@V-In_2O_3, and Cl@V-In_2O_3.

Bond	V-In_2O_3	P@V-In_2O_3	S@V-In_2O_3	Cl@V-In_2O_3
N-N	-12.09774	-9.9054	-12.06698	-12.07621
N-In	-0.29862	-1.4307	-0.23747	-0.2063
N-O/S/P/Cl	-0.00687	-4.6619	-0.00169	-0.00151
